# Trends and Disparities in Pneumothorax‐Related Deaths in the United States Before and During the COVID‐19 Pandemic: A CDC WONDER Database Analysis

**DOI:** 10.1002/puh2.70322

**Published:** 2026-07-31

**Authors:** Mariam Idrees, Eman Imtisal, Warda Fatima Shafee, Muddassir Khalid, Taha Mazhar Awan, Syeda Fatima Harrum Bukhari, Fatima Yasmeen Awan, Fatima Idrees, Sumia Fatima, Rida Noor, Asad Khan, Fred Segawa

**Affiliations:** ^1^ Foundation University Medical College Islamabad Pakistan; ^2^ Nishtar Medical University Multan Pakistan; ^3^ Islamic International Medical College Riphah International University Rawalpindi Pakistan; ^4^ Rawalpindi Medical University Rawalpindi Pakistan; ^5^ Bolan Medical College Quetta Pakistan; ^6^ Bacha Khan Medical College Mardan Pakistan; ^7^ Makerere University College of Health Sciences, Central Region Kampala Uganda

**Keywords:** Centers for Disease Control and Prevention (CDC), COVID‐19 pandemic, mortality trends, pneumothorax, regional and ethnic disparities

## Abstract

**Background:**

There was an increase in pneumothorax‐related deaths in the United States during the COVID‐19 pandemic era, likely due to the load on the healthcare system and as a complication following the viral infection itself.

**Aims:**

To analyze the mortality trends, variations, and most affected demographic subgroups in pneumothorax‐related deaths before and during the COVID‐19 pandemic.

**Methods:**

A retrospective‐cohort‐study was conducted using patient data, which was collected using the Centers for Disease Control and Prevention Wide‐Ranging Online Data for Epidemiological Research (CDC WONDER) database for deaths from pneumothorax before (1999–2019) and during the COVID‐19 pandemic (2020–2021). Age‐adjusted mortality rates (AAMRs) were calculated per 100,000, and analysis was performed by Joinpoint, which estimated average annual‐percentage‐change (AAPC).

**Results:**

A total of 74,799 deaths occurred from pneumothorax from 1999 to 2021. Pneumothorax mortality rates were low before the pandemic (AAPC, −0.306 and AAMR, 0.8) but reversed during the pandemic (AAMR, 2.15; AAPC, 79.94). Overall, during the pandemic, the AAMRs were greater for men, NH‐American Indians/Alaska Natives, the Southern region, and adults aged ≥65 years. Mortality rates varied regionally, with the highest in Kentucky (4.1) and the lowest in Maine (0.5). States like South Carolina, Tennessee, Wyoming, Texas, Kentucky, and West Virginia fell into the 90th percentile. The sharpest increase was observed among American Indian people (AAPC, 169.74), non‐Hispanic Black people (AAPC, 64.82), Hispanic people (AAPC, 151.67), younger adults (AAPC, 156.19), the Southern region (AAPC, 70), females (AAPC, 78.78), and older adults (AAPC, 56.414) for pneumothorax‐related mortality during the pandemic.

**Conclusions:**

As pneumothorax was identified using multiple cause‐of‐death records, these findings should be interpreted as describing mortality patterns in which pneumothorax contributed to death rather than establishing pneumothorax as the primary cause. Continued surveillance and further studies incorporating clinical‐level data are warranted to better understand the drivers of these observed trends.

## Graphical abstract

Central illustration for mortality trends of respiratory disorders in obese patients among the US population from 1999 to 2023: A CDC WONDER analysis.

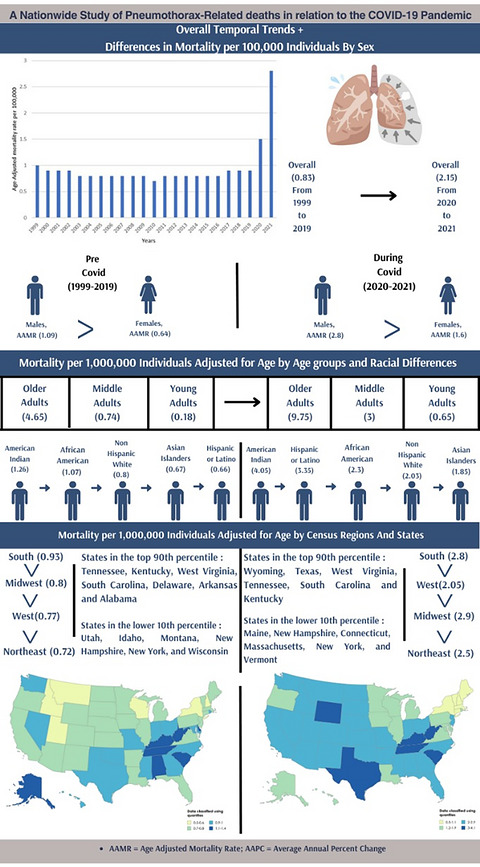

## 1 Introduction

Pneumothorax is the accumulation of air within the pleural cavity, outside the lung. This condition occurs when air accumulates between the parietal and visceral pleurae in the chest, exerting pressure on the lung and potentially leading to its collapse [1]. A study conducted in Minnesota reported the age‐adjusted incidence of spontaneous pneumothorax between 1950 and 1974 as 7.4 and 1.2 per 100,000 per year for males and females, respectively, for primary pneumothorax and 6.3 and 2.1/100,000/year for secondary pneumothorax. The ratio of male‐to‐female incidence was 6.2:1 for primary and 3.2:1 for secondary spontaneous pneumothorax. There was no significant difference in the incidence rates of primary and secondary pneumothorax by sex or overall [2]. This topic requires review and analysis because, despite the lack of solid data, mortality rates appear to have increased during the COVID‐19 pandemic.

It is reported that healthcare systems worldwide were profoundly affected by the COVID‐19 pandemic, not only through the direct burden of the infection but also by causing widespread disruption to routine healthcare delivery [3]. Acute respiratory distress syndromes, pleural diseases like effusion and pneumothorax, and self‐limiting respiratory infections were among the many respiratory system complications brought on by the COVID‐19 infection that ultimately led to acute respiratory failure [4, 5]. The primary cause of mortality due to COVID‐19 is the respiratory compromise; however, pneumothorax appears to be a secondary complication of the infection. Although the majority of patients required noninvasive ventilation, a significant proportion required intubation and invasive mechanical ventilation, which hindered their complete recovery [6]. Pneumothorax also appeared to be a fatal complication in patients who required mechanical ventilation [7], with a reported incidence of up to 15% [8], requiring surgery for complete recovery [9]. It suggests that the number of deaths from pneumothorax increased significantly during the pandemic. Although numerous clinical studies have described pneumothorax as a complication of COVID‐19 and mechanical ventilation, most have been limited to single‐center cohorts, case series, or short‐term observational studies. National population‐level analyses evaluating long‐term mortality trends and demographic disparities remain relatively limited [10, 11, 12, 13, 14]. An epidemiological study found that the absolute change in the incidence of cases reporting pneumothorax was minimal (between the pre‐pandemic era and the first wave of lockdown); however, higher crests were found in the second and third waves of lockdown, with a peak in January 2021, reporting an incidence rate ratio 1.65 times higher than the pre‐pandemic era [15]. These data are from European retrospective analyses.

This study used mortality data from the CDC (Centers for Disease Control and Prevention) database to describe the national trends in pneumothorax‐related mortality in the United States before and during the pandemic. Although increased mortality during the pandemic has been reported, this study provides a comprehensive population‐level analysis of long‐term trends, demographic disparities, and geographic variation, offering insights beyond the immediate impact of COVID‐19.

## 2 Methodology

### 2.1 Study Design

In this study, a retrospective observational design was employed using mortality data from the CDC Wide‐Ranging Online Data for Epidemiological Research (WONDER) database to evaluate trends in pneumothorax‐related deaths in the United States. The study population included all individuals with pneumothorax listed as a cause of death on death certificates, identified using International Classification of Diseases, 10th Revision (ICD‐10) codes (J93.0, J93.1, J93.8, and J93.9). The database provides nationally representative mortality data, including demographic characteristics such as age, sex, race/ethnicity, and geographic location. This study has been reported in line with STROBE guidelines checklist.

### 2.2 Data Sources

Data from the CDC's WONDER database were used to assess trends in pneumothorax‐related fatalities in the pre‐pandemic period (1999–2019) and during the COVID‐19 pandemic (2020–2021). We used the following ICD‐10 codes, listed as multiple causes of death, to identify deaths from pneumothorax: J93.0, J93.1, J93.8, and J93.9 [16, 17, 18, 19]. Pneumothorax‐related deaths were identified using ICD‐10 codes listed as multiple causes of death on death certificates, rather than restricting to the underlying cause of death. This approach was chosen to capture the broader contribution of pneumothorax to mortality, particularly in complex clinical scenarios, such as COVID‐19 infection, where pneumothorax may act as a contributing rather than primary cause.

### 2.3 Statistical Analysis

Death certificate data were recorded on population size, year, and demographic characteristics, including sex, race and ethnicity, age, and census regions. Race and ethnicity categories included NH‐White, NH‐Black, Hispanic/Latino, NH‐American Indian/Alaskan Native, and NH‐Asian/Pacific Islander. Age‐adjusted mortality rates (AAMRs) per 100,000 people were calculated and grouped by sex, race, ethnicity, and age. In the pneumothorax population, deaths among individuals aged <25 years were excluded to minimize potential misclassification related to congenital, traumatic, or neonatal causes of pneumothorax, which differ significantly in etiology and clinical course from adult spontaneous and secondary pneumothorax. This restriction was applied to improve the homogeneity and interpretability of the study population. For race‐stratified analyses, limited missing year‐specific values were encountered. These were handled using mean substitution to preserve continuity in trend visualization; however, given the potential for bias with this approach, results were interpreted cautiously, and this limitation is acknowledged. Trend‐level analysis was performed using the Joinpoint Regression Program version 5.1.0.0 (National Cancer Institute), which estimates average annual‐percentage‐change (AAPC) in AAMR with a corresponding 95% CI. AAPC in AAMR was calculated to identify trends in AAMR for the years preceding the COVID‐19 pandemic (1999–2019) and the years during the pandemic (2020–2021). A two‐sided significance level of *p *< 0.05 was considered statistically significant. This study was exempt from institutional review board approval, as all data used are publicly available and de‐identified.

## 3 Results

### 3.1 Overall Trends

**FIGURE 1:**
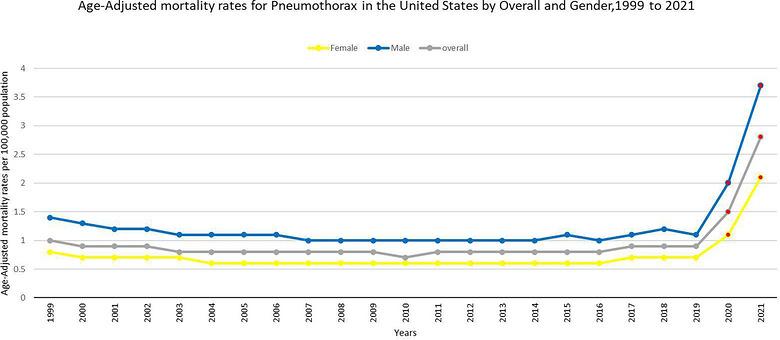
Age‐adjusted mortality rates for pneumothorax‐related deaths in the United States stratified by overall and sex between 1999 and 2021.

A total of 74,799 deaths occurred from 1999 to 2021. The AAMR decreased slightly from 1 per 100,000 in 1999 to 0.9 in 2019 (AAPC, −0.306 [95% CI, −0.842 to 0.252]; Figure 1), *p* value (0.268). This downward trend reversed during the COVID‐19 pandemic, with AAMR increasing to 1.5 in 2020 and 2.8 in 2021 (total AAMR, 2.15; AAPC, 79.942 [95% CI, 20.552–168.591], *p* value (0.034); Tables S1 and S2).

### 3.2 Sex

Men had higher AAMR than women before the pandemic, although both had a similar downward trend (men: AAMR, 1.095; AAPC: −0.909 [95% CI, −1.71 to −1.95]; *p* value (0.020); women: AAMR, 0.64; AAPC: −0.49 [95% CI, −1.19 to 0.19]; *p* value (0.149); (Figure 1). During the pandemic, men still had greater AAMR than women (AAMR, 2.8 and 1.6, respectively) (men: AAPC, 83.03 [95% CI, 72.97–95.80], *p* value (0.005); women: AAPC, 78.78 [95% CI, −9.27 to 252.30], *p* value (0.058); Tables S1 and S2).

### 3.3 Race and Ethnicity

**FIGURE 2:**
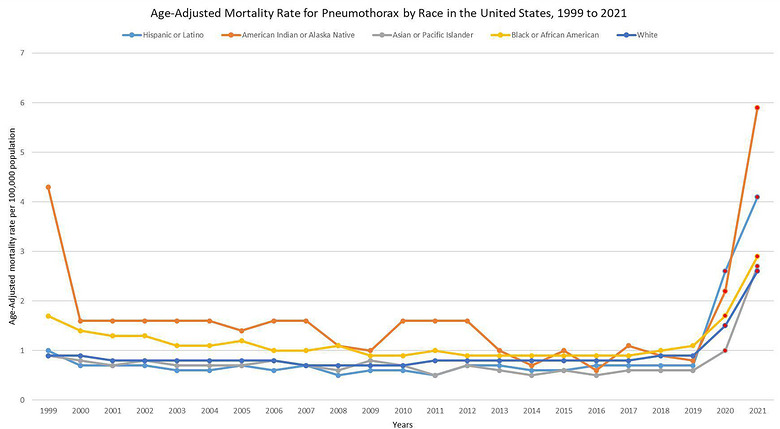
Age‐adjusted mortality rates for pneumothorax‐related deaths in the United States stratified by race/ethnicity between 1999 and 2021.

American Indian people had the highest AAMRs both before (AAMR, 1.26; AAPC, −3.26 [95% CI, −6.94 to 0.55]; *p* value (0.083)) and during the pandemic (AAMR, 4.05; AAPC, 169.74 [95% CI, 144.21–197.92]; *p* value (0.005)), compared with African American people (pre‐pandemic AAMR, 1.07; AAPC, −0.0048 [95% CI, −0.062 to 0.053]; *p* value (0.865); pandemic AAMR, 2.3; AAPC, 64.82 [95% CI, 15.99–134.21]; *p* value (0.035)) and Hispanic people who had a significant increase (pre‐pandemic AAMR, 0.66; AAPC, 2.674 [95% CI, 1.82–3.52]; *p* value (0.000); pandemic AAMR, 3.35; AAPC, 151.67 [95% CI, −47.54 to 1107.45]; *p* value (0.084)). Non‐Hispanic White people also witnessed a significant rise (pre‐pandemic AAMR, 0.8; AAPC, 0.023 [95% CI, −0.548 to 0.597] *p* value (0.933); pandemic AAMR, 2.05; AAPC, 71.1173 [95% CI, 48.645–96.98] *p* value (0.013)). During the pandemic, all categories had a comparable increase in mortality rate except non‐Hispanic Asian or Pacific Islander people, who had a relatively minor increase (pre‐pandemic AAMR, 0.67; AAPC, −1.95 [95% CI, −2.80 to 1.09]; *p* value (0.000); pandemic AAMR, 1.85; AAPC, 64.82 [95% CI, 48.64–98.96]; *p* value (0.152); Figure 2, Tables S3 and S4).

### 3.4 Age

**FIGURE 3:**
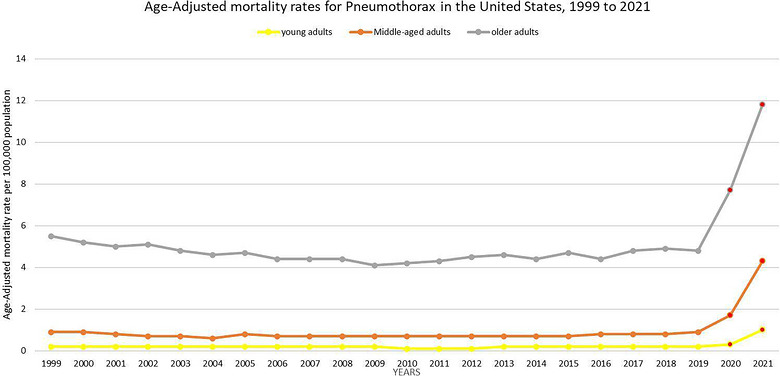
Age‐adjusted mortality rates for pneumothorax‐related deaths in the United States stratified by age group between 1999 and 2021.

The highest AAMRs were observed in older adults (aged ≥65 years) both before (AAMR, 4.65; AAPC, −0.536 [95% CI, −1.029 to −0.041]; *p* value (0.035)) and during the pandemic (AAMR, 9.75; AAPC, 56.414 [95% CI, 53.24–59.91]; *p* value (<0.000)), compared with other age groups (Figure 3). A significant upward trend during the pandemic was also seen in both young adults aged 25–44 years (AAMR, 0.65; AAPC, 156.19 [95% CI, 115.11–469.09]; *p* value (<0.000)) and middle‐aged adults aged 45–64 years (AAMR, 3; AAPC, 88.89 [95% CI, 88.59–89.16]; *p* value (<0.000)) (Tables S5 and S6).

### 3.5 Geographical/Census Regions

**FIGURE 4:**
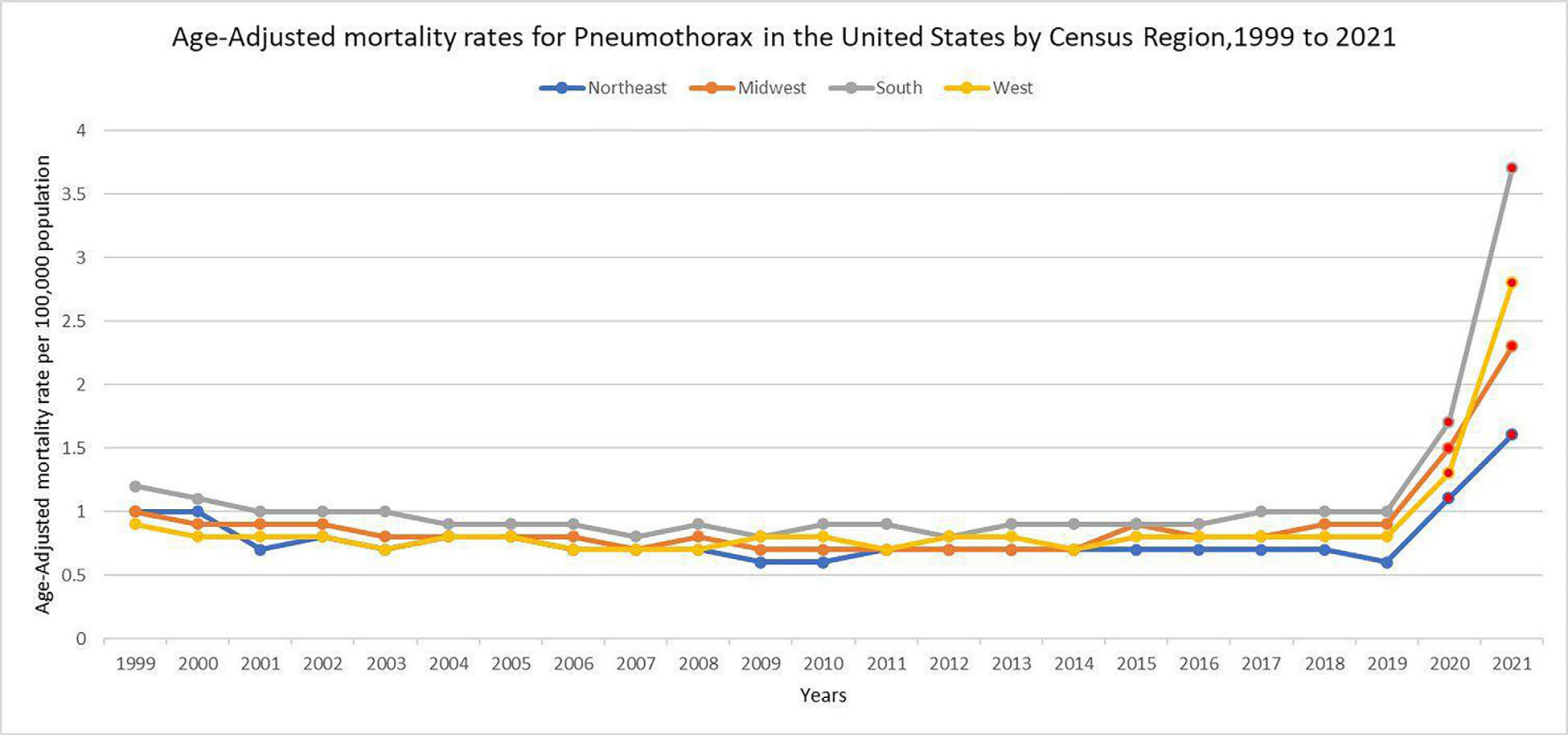
Age‐adjusted mortality rates for pneumothorax‐related deaths in the United States stratified by census regions between 1999 and 2021.

During the pre‐pandemic era, the highest AAMR was noted in the Southern region at 0.93 (AAPC, −0.66 [95% CI, −1.35 to 0.027]), *p* value (0.058), followed by the Midwestern region at 0.80 (AAPC, −0.13 [95% CI, −0.63 to 0.37]), *p* value (0.209), the Western region at 0.77 (AAPC, −0.48 [95% CI, −1.26 to 0.29]), *p* value (0.597), and the Northeast region at 0.72 (AAPC, −1.70 [95% CI, −2.48 to −0.92]), *p* value (0.000) (Figure 4). Although the AAMR of each state increased significantly during the pandemic, the trend also changed slightly as compared to the pre‐pandemic era, with the highest AAMRs seen in the Southern region at 2.7 (AAPC, 70 [95% CI, 69.97–70.02]); *p* value (0.000), followed by the Western region at 2.05 (AAPC, 62.5 [95% CI, 62.47–62.52]), *p* value (0.000), the Midwestern region at 1.9 (AAPC, 66.667 [95% CI, 66.66–66.67]); *p* value (0.000), and the Northeast region at 1.35 (AAPC, −0.536 [95% CI, −1.029 to −0.041]); *p* value (0.097) (Tables S7 and S8).

### 3.6 States

**FIGURE 5:**
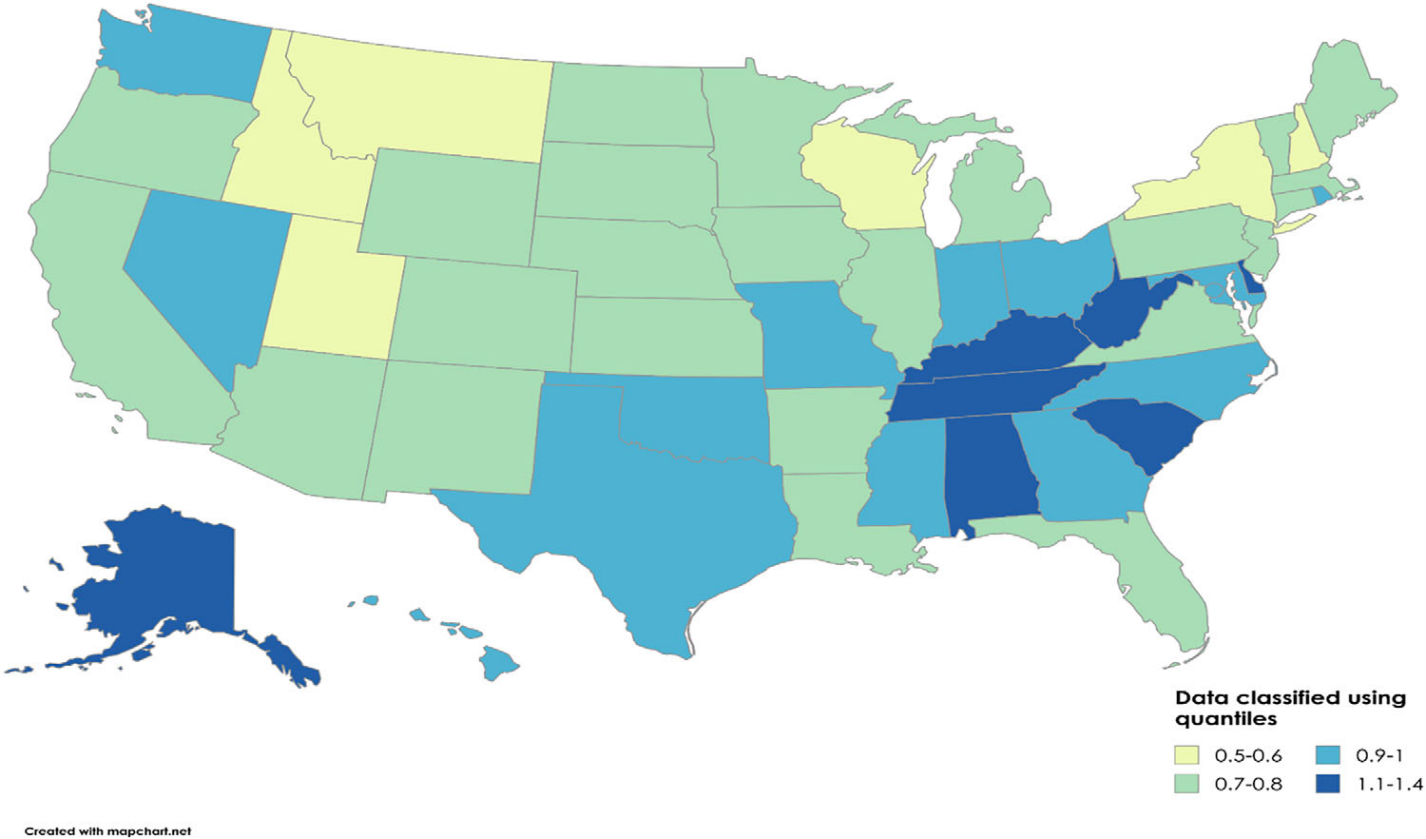
Age‐adjusted mortality rates for pneumothorax‐related deaths in the United States stratified by states, before COVID‐19.

**FIGURE 6:**
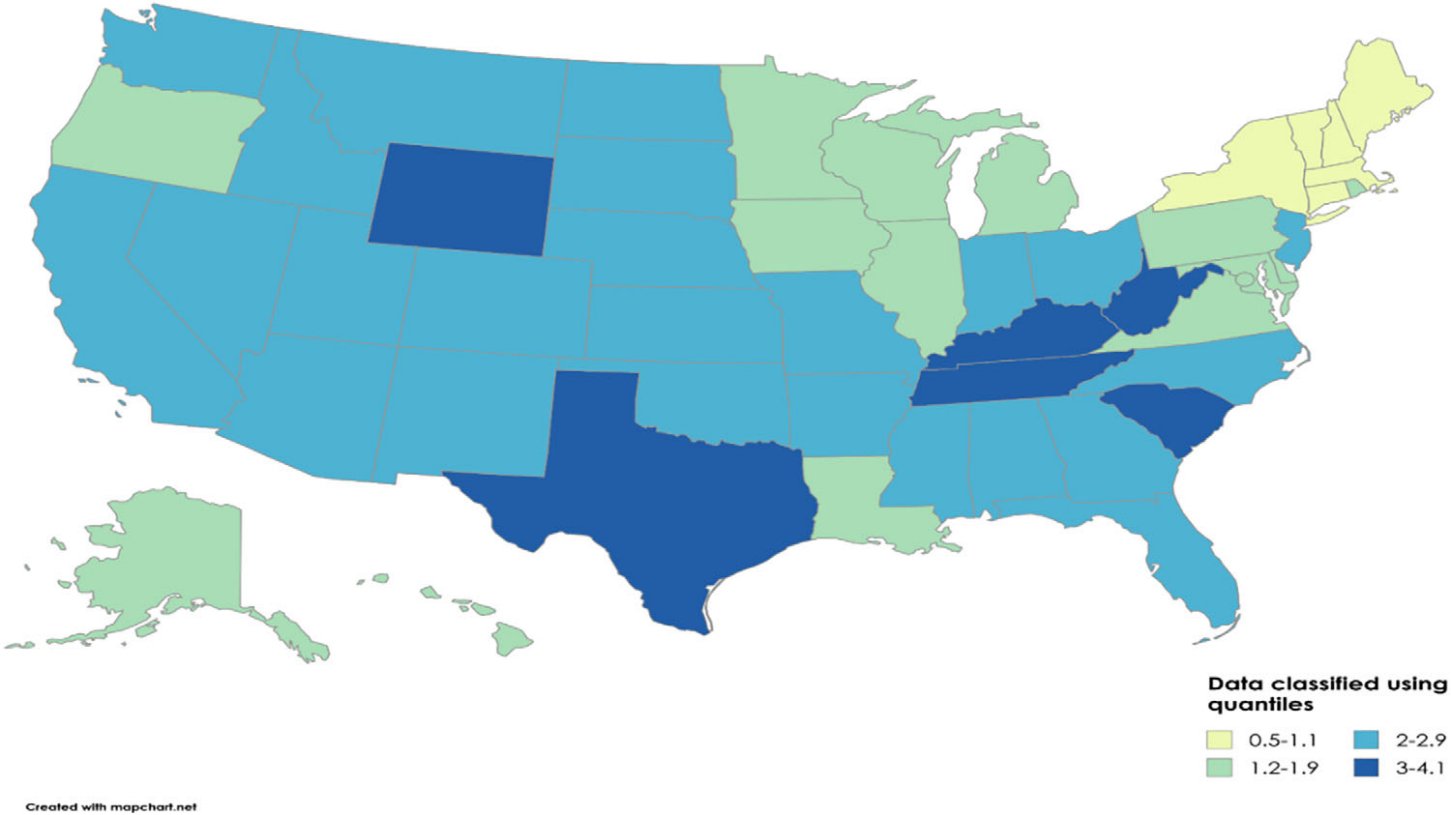
Age‐adjusted mortality rates for pneumothorax‐related deaths in the United States stratified by states, after COVID‐19.

In the pre‐pandemic era, the highest AAMR was reported by West Virginia at 1.4 (95% CI: 1.3–1.5), and Utah reported the lowest AAMR at 0.5 (95% CI: 0.5–0.6). States like West Virginia (1.4), Kentucky (1.3), Tennessee (1.2), South Carolina (1.1), Delaware (1.1), Arkansas (1.1), and Alabama (1.1) fell into the top 90th percentile of the AAMRs, whereas Utah (0.5), Idaho (0.6), Montana (0.6), New Hampshire (0.6), New York (0.6), and Wisconsin (0.6) had their AAMRs in the lower 10th percentile. During the pandemic era, the highest AAMR was reported in Kentucky at 4.1 (95% CI: 3.7–4.5), and the lowest AAMR was reported in Maine at 0.5 (95% CI: 0.3–0.8). States, such as Kentucky (4.1), Texas (3.9), Wyoming (3.3), West Virginia (3.2), Tennessee (3.2), and South Carolina (3), fell into the top 90th percentile of the AAMRs, whereas Maine (0.5), New Hampshire (1), Connecticut (1.1), Massachusetts (1.1), New York (1.1), and Vermont (1.1) had their AAMRs in the lower 10th percentile (Table S9, Figures 5 and 6).

## 4 Discussion

This study analyzed national mortality data on deaths from pneumothorax that occurred both before and during the COVID‐19 pandemic. Before the COVID‐19 pandemic, pneumothorax mortality exhibited a modest declining trend in the mortality rates, with AAMR stating 0.8 and AAPC −0.30; however, the course shifted considerably during the pandemic, with the AAMR rising to 2.15 and AAPC increasing sharply to 79.94. Kentucky, Texas, Wyoming, West Virginia, Tennessee, and South Carolina fell into the top 90th percentile of pneumothorax‐related deaths during the COVID‐19 pandemic, whereas Maine, New Hampshire, Connecticut, Massachusetts, New York, and Vermont fell into the lower 10th percentile. Observational analysis revealed that not all US states responded to the COVID‐19 pandemic with equal efficiency. These disparities in response effectiveness were strongly associated with underlying racial biases, health disparities, and inconsistent policy implementation that eventually led to poor vaccination rates across some states [20]. As the current analysis is limited to data up to 2021, it is not possible to definitively determine whether pneumothorax‐related mortality has returned to pre‐pandemic levels. However, emerging post‐pandemic epidemiological reports suggest a gradual normalization of healthcare utilization and critical care burden, which may influence future mortality trends. Further studies incorporating more recent data are needed to evaluate whether these elevated mortality rates persist. The gradual decline in pneumothorax‐related mortality observed in the pre‐pandemic period may reflect improvements in clinical management, including advancements in imaging, early diagnosis, minimally invasive thoracic interventions, and standardized treatment protocols. Increased awareness and improved access to healthcare services may also have contributed to better outcomes over time. The reversal of this trend during the pandemic underscores the impact of systemic healthcare disruption.

Pre‐pandemic geographic variation in pneumothorax mortality may be attributed to differences in healthcare infrastructure, availability of specialized thoracic care, population health status, and socioeconomic disparities across states. Variability in reporting practices and regional disease burden may also contribute to these differences.

Overall, temporal trends appeared largely consistent across demographic subgroups; however, we observed differences in the magnitude of mortality rates by race, ethnicity, gender, and age, which became more pronounced after the onset of the COVID‐19 pandemic. A critical consideration in interpreting these findings is that AAMR reflects absolute mortality levels, whereas AAPC captures the relative rate of change in AAMR. Thus, although AAPCs are informative for evaluating temporal patterns within a given subgroup, they are not directly comparable across subgroups with markedly different baseline mortality rates, as such comparisons may distort the actual differences in disease burden [3].

Numerous investigations have demonstrated that pneumothorax became an increasingly recognized complication of COVID‐19, particularly among patients with severe pneumonia and those requiring invasive mechanical ventilation. Multicenter studies by Martinelli et al., Miro et al., Chopra et al., Zantah et al., and Geraci et al. consistently reported increased morbidity and mortality among patients who developed pneumothorax during COVID‐19 infection [21, 22, 23, 24, 25]. Proposed mechanisms include diffuse alveolar injury, inflammatory destruction of lung parenchyma, barotrauma associated with mechanical ventilation, and increased pulmonary frailty. Our findings complement these clinical investigations by demonstrating that the impact of pneumothorax extended to measurable increases in population‐level mortality during the pandemic [24, 25].

Various studies have found pneumothorax as a subsequent complication to COVID‐19 infection and an eventual cause of fatality, even in patients without a history of mechanical ventilation [26, 27]. The incidence of pneumothorax increases significantly in patients with severe and prolonged COVID‐19 infection who end up requiring invasive ventilation procedures, and it should be considered as a potential life‐threatening sequela of COVID‐19 [4]. Retrospective analysis of medical records, laboratory findings, chest x‐rays, and CT images of patients complicated by pneumothorax during active COVID‐19 revealed that pneumothorax most likely developed due to alveolar wall destruction, and some also reported the presence of free air and lesions in lungs [28, 29].

We found AAMR to be higher in males than in women; however, both genders shared a rise in mortality rates during the pandemic. Data from the studies showed that despite having the same susceptibility, males were more prone to subsequent COVID‐19 complications and death than females, independent of age [30]. It has been associated in multiple studies with female hormones, mainly estrogen and progesterone, which are referred to as immunomodulators. Primarily, they inhibit pro‐inflammatory cytokines and have also been found to have antiviral properties, thereby boosting both the innate and adaptive immune responses [31, 32, 33]. Females tend to exhibit a more effective and stronger immune response to infectious diseases than males. This mechanism can be primarily linked to the presence of two X chromosomes, which carry several immune‐related genes [34].

In our data analysis, we found the highest number of deaths due to pneumothorax in the Southern region, followed by the lowest in the Northeast region. Studies indicate that stark disparities in COVID‐19 mortality rates first emerged during the summer of 2020 in the South and Northeast areas, well before vaccines were widely available. In fact, 63% of the avoidable deaths had already occurred in the South by the end of February 2021, when the vaccine distribution phase was just beginning [35]. The highest rates of COVID‐19 cases and deaths were observed in the Southern region, which can be attributed to flaws in political policies, lower socioeconomic position, inadequate adherence to SOPs during COVID, and restricted access to healthcare facilities [36], thus a higher likelihood of complications as sequelae to COVID‐19 infection in that specific region.

Racial biases and inequities influence employment and education opportunities, affecting the future. Racial/ethnic minorities have reported decreased life expectancy during COVID‐19 due to gaps in social determinants and systemic inequities [37]. American Indian/Alaska Native people faced the highest rate of mortality and Non‐Hispanic due to pneumothorax during the pandemic era. In one study, it was reported that American Indian/Alaska Native ethnicity experienced a higher mortality rate during the pandemic than any other ethnic/race group and about eight times more than the White population [38]. Racial minorities faced a significant number of hospitalizations, complications, and deaths during the pandemic, mainly due to disparities in insurance, vaccination rates, healthcare access, and exposure rates [32, 39, 40, 41]. These disparities may also reflect structural inequities, including differences in access to timely healthcare, baseline comorbidity burden, occupational exposure risk, and healthcare system biases. Additionally, variations in vaccination uptake and healthcare‐seeking behavior during the pandemic may have further amplified these differences.

Older adults remain particularly vulnerable because advanced age is associated with diminished pulmonary reserve, increased comorbidity burden, greater likelihood of mechanical ventilation, and reduced physiological resilience following respiratory complications. This increase can be linked to the presence of comorbidities, age‐related lung deterioration, and reduced immunity, leading to a greater risk of developing complications like pneumothorax during the pandemic [42, 43, 44, 45].

Future investigations should incorporate linked hospitalization databases, patient‐level clinical information, and longitudinal follow‐up to distinguish pneumothorax as an underlying versus contributing cause of death and to evaluate whether mortality patterns observed during the COVID‐19 pandemic have returned to pre‐pandemic levels. Such studies will further clarify the mechanisms underlying pneumothorax‐associated mortality and inform future prevention strategies [46, 47].

## 5 Limitations

Several limitations should be acknowledged when interpreting our findings.

First, pneumothorax‐related deaths were identified using multiple cause‐of‐death coding rather than restricting analyses to the underlying cause of death. Consequently, pneumothorax may have represented a contributing complication rather than the principal cause of death in some patients, particularly during the COVID‐19 pandemic when severe respiratory failure frequently coexisted with pneumothorax. Therefore, our findings should be interpreted as describing mortality patterns associated with pneumothorax rather than direct pneumothorax‐specific mortality.

The use of mortality data alone does not allow estimation of case‐fatality rates, as linkage to hospital admission data was not available within the CDC WONDER database. Therefore, it was not possible to calculate mortality per hospitalization, which may provide a more clinically relevant measure of risk.

A key limitation of this study is the inability to stratify pneumothorax cases by etiology (e.g., primary spontaneous, secondary, traumatic, or ventilator‐associated pneumothorax). This distinction is particularly relevant during the COVID‐19 pandemic, where increased use of mechanical ventilation may have contributed to higher rates of secondary pneumothorax. The absence of etiological differentiation limits the ability to determine whether observed mortality trends reflect true epidemiological changes or shifts in clinical context.

Additionally, relying on death certificate data limited our understanding of how diagnoses were determined, particularly in non‐healthcare settings. This raises the possibility of overlap between deaths caused solely by pneumothorax and those involving pneumothorax in the context of a COVID‐19 infection. Another potential limitation is that the database lacked socioeconomic variables, which limited the ability to adjust for income, insurance coverage, and related disparities that may underlie subgroup differences in mortality. Specific data disparities were also found, such as the data for the American race (for the years 2001 and 2002) and urbanization (2020 and 2021), which were missing. The data for the Native Hawaiian race (2021 and 2022) and the American Indian race (2000, 2003, 2004, 2006, 2007, 2010, 2011, and 2012) were deleted because they were labeled as unreliable on the CDC database. Lastly, the data for the age group less than 25 was not included because of the presence of some birth‐related issues or misinterpretation of the cause of death.

## 6 Conclusions

This nationwide analysis demonstrated significant temporal, demographic, and geographic variation in pneumothorax‐related mortality in the United States, including a marked increase during the COVID‐19 pandemic. However, because deaths were identified using multiple cause‐of‐death coding, these findings should be interpreted as describing associations between pneumothorax and mortality rather than indicating pneumothorax as the underlying cause of death. Although the observed trends may reflect evolving disease patterns, healthcare delivery, and the impact of COVID‐19, additional studies using patient‐level clinical data are needed to clarify causal mechanisms and evaluate post‐pandemic mortality trends.

## Author Contributions

**Mariam Idrees**: conceptualization, project administration, data curation, methodology, writing – original draft preparation. **Eman Imtisal**: conceptualization, writing – original draft preparation, data curation, formal analysis. **Taha Mazhar Awan**: data curation, visualization, validation, software. **Syeda Fatima Harrum Bukhari**: data curation, formal analysis. **Fatima Yasmeen Awan**: data curation, writing – review and editing. **Fatima Idrees**: formal analysis, validation. **Rida Noor**: investigation, methodology, writing – review and editing. **Asad Khan**: formal analysis, writing – review and editing. **Sumia Fatima**: validation, visualization, writing – review and editing. **Fred Segawa**: validation, visualization, writing – review and editing. **Muddassir Khalid**: supervision, project administration, writing – review and editing. All authors read and approved the final manuscript and consented to the submission of this manuscript.

## Funding

The authors have nothing to report.

## Ethics Statement

The authors have nothing to report.

## Consent

The authors have nothing to report.

## Conflicts of Interest

The authors declare no conflicts of interest.

## Supporting information




**Supporting File 1**: puh270322‐sup‐0001‐SuppMat.docx

## Data Availability

This study used anonymized, publicly available data from the Centers for Disease Control and Prevention Wide‐Ranging Online Data for Epidemiologic Research (CDC WONDER) (https://wonder.cdc.gov/mcd.html).
