# Supplementary material for: Trends and Disparities in Pneumothorax‐Related Deaths in the United States Before and During the COVID‐19 Pandemic: A CDC WONDER Database Analysis
Source: Public Health Chall. 2026 Jul 31;5(3):e70322. doi: 10.1002/puh2.70322 (PMC13426481; doi:10.1002/puh2.70322)
Supplement: Supplementary file 1 — Supporting File 1: puh270322‐sup‐0001‐SuppMat.docx [file PUH2-5-e70322-s001.docx]

**SUPPLEMENTAL FILES**

**Table S1: Pneumothorax- Related Age-adjusted mortality rate for Overall, Men, Women in the United States before and during the COVID-19 pandemic.**

|  | **Age-Adjusted Rate (95% CI)** | | |
| --- | --- | --- | --- |
| **Year** | **Overall** | **Males** | **Women** |
| **1999** | 1  (1-1.1) | 1.4 (1.4–1.5) | 0.8 (0.7–0.8) |
| **2000** | 0.9  (0.9 - 1) | 1.3 (1.3–1.4) | 0.7 (0.7–0.7) |
| **2001** | 0.9  (0.8 - 0.9) | 1.2 (1.1–1.2) | 0.7 (0.7–0.7) |
| **2002** | 0.9  (0.9 - 0.9) | 1.2 (1.1–1.2) | 0.7 (0.6–0.7) |
| **2003** | 0.8  ( 0.8 - 0.9) | 1.1 (1.0–1.2) | 0.7 (0.6–0.7) |
| **2004** | 0.8  (0.8 - 0.8) | 1.1 (1.0–1.2) | 0.6 (0.6–0.6) |
| **2005** | 0.8  ( 0.8 - 0.9) | 1.1 (1.1–1.2) | 0.6 (0.6–0.7) |
| **2006** | 0.8  (0.7 - 0.8) | 1.1 (1.0–1.1) | 0.6 (0.5–0.6) |
| **2007** | 0.8  (0.7 - 0.8) | 1.0 (1.0–1.1) | 0.6 (0.6–0.6) |
| **2008** | 0.8  (0.7 - 0.8) | 1.0 (1.0–1.1) | 0.6 (0.6–0.6) |
| **2009** | 0.8  (0.7 - 0.8) | 1.0 (0.9–1.0) | 0.6 (0.6–0.6) |
| **2010** | 0.7   (0.7 - 0.8) | 1.0 (0.9–1.0) | 0.6 (0.5–0.6) |
| **2011** | 0.8  (0.7 - 0.8) | 1.0 (1.0–1.1) | 0.6 (0.5–0.6) |
| **2012** | 0.8  (0.8 - 0.8) | 1.0 (1.0–1.1) | 0.6 (0.6–0.6) |
| **2013** | 0.8  (0.8 - 0.8) | 1.0 (1.0–1.1) | 0.6 (0.6–0.6) |
| **2014** | 0.8  (0.7 - 0.8) | 1.0 (1.0–1.1) | 0.6 (0.6–0.6) |
| **2015** | 0.8  (0.8 - 0.9) | 1.1 (1.0–1.1) | 0.6 (0.6–0.7) |
| **2016** | 0.8  (0.8 - 0.8) | 1.0 (1.0–1.1) | 0.6 (0.6–0.7) |
| **2017** | 0.9  (0.8 - 0.9) | 1.1 (1.0–1.1) | 0.7 (0.6–0.7) |
| **2018** | 0.9  (0.9 - 0.9) | 1.2 (1.1–1.2) | 0.7 (0.6–0.7) |
| **2019** | 0.9  (0.9 - 0.9) | 1.1 (1.1–1.2) | 0.7 (0.7–0.7) |
| **Pre-COVID Average AAMR** | 0.833  (0.78- 0.86) | 1.1 (1.0–1.2) | 0.6 (0.6–0.7) |
| **2020** | 1.5  (1.4 - 1.5) | 2.0 (1.9–2.1) | 1.1 (1.0–1.1) |
| **2021** | 2.8  (2.8 - 2.9) | 3.7 (3.6–3.8) | 2.1 (2.0–2.2) |
| **During COVID-19 Average AAMR** | 2.15  (2.1 - 2.2) | 2.85 (2.8–3.0) | 1.6 (1.5–1.7) |

**Table S2:** Pneumothorax-related Average Annual Percentage Rate for Overall, Men, and Women in the United States before and during the COVID-19 pandemic.

| **Cohort** | **Range** | **AAPC** | **LOWER CI** | **UPPER CI** |  |
| --- | --- | --- | --- | --- | --- |
| **Overall** | 1999- 2019 | -0.3065 | -0.842 | 0.2525 |  |
| **Overall** | 2019- 2021 | 79.9426* | 20.5523 | 168.5918 |  |
| **Women** | 1999- 2019 | -0.493 | -1.1931 | 0.1921 |  |
| **Women** | 2019- 2021 | 78.7795 | -9.2766 | 252.3028 |  |
| **Men** | 1999- 2019 | -0.9095* | -1.711 | -0.1955 |  |
| **Men** | 2019- 2021 | 84.0343* | 72.9742 | 95.8016 |  |

**NOTE: asterisk indicates a p-value of < 0.05**

**Table S3: Pneumothorax-related Age-Adjusted mortality rate for NH Black or African American, NH White, NH Asian Pacific, NH American Indians, and Hispanic or Latino in the United States before and during the COVID-19 pandemic**

|  | **Age-Adjusted Rate (95% CI)** | | | | |
| --- | --- | --- | --- | --- | --- |
| **Year** | **American Indian** | **Asians and Pacifics** | **Black or African** | **White** | **Hispanic or Latino** |
| **1999** | 4.3 (3.1–5.8) | 0.9 (0.7–1.2) | 1.7 (1.5–1.8) | 0.9 (0.9–1.0) | 1.0 (0.8–1.1) |
| **2000** |  | 0.8 (0.6–1.1) | 1.4 (1.3–1.6) | 0.9 (0.9–0.9) | 0.7 (0.6–0.8) |
| **2001** |  | 0.7 (0.6–1.0) | 1.3 (1.2–1.4) | 0.8 (0.8–0.9) | 0.7 (0.5–0.8) |
| **2002** |  | 0.8 (0.6–1.0) | 1.3 (1.2–1.4) | 0.8 (0.8–0.9) | 0.7 (0.5–0.8) |
| **2003** |  | 0.7 (0.5–0.9) | 1.1 (1.0–1.2) | 0.8 (0.8–0.9) | 0.6 (0.5–0.7) |
| **2004** |  | 0.7 (0.5–0.9) | 1.1 (1.0–1.3) | 0.8 (0.7–0.8) | 0.6 (0.5–0.8) |
| **2005** | 1.4 (0.9–2.2) | 0.7 (0.5–0.9) | 1.2 (1.0–1.3) | 0.8 (0.8–0.8) | 0.7 (0.6–0.8) |
| **2006** |  | 0.8 (0.6–1.0) | 1.0 (0.9–1.1) | 0.8 (0.7–0.8) | 0.6 (0.5–0.8) |
| **2007** |  | 0.7 (0.5–0.9) | 1.0 (0.9–1.1) | 0.7 (0.7–0.8) | 0.7 (0.6–0.8) |
| **2008** | 1.1 (0.7–1.8) | 0.6 (0.5–0.8) | 1.1 (1.0–1.2) | 0.7 (0.7–0.8) | 0.5 (0.4–0.6) |
| **2009** | 1.0 (0.6–1.6) | 0.8 (0.6–1.0) | 0.9 (0.8–1.0) | 0.7 (0.7–0.8) | 0.6 (0.5–0.7) |
| **2010** |  | 0.7 (0.5–0.9) | 0.9 (0.8–1.0) | 0.7 (0.7–0.8) | 0.6 (0.5–0.7) |
| **2011** |  | 0.5 (0.4–0.6) | 1.0 (0.9–1.1) | 0.8 (0.7–0.8) | 0.5 (0.4–0.6) |
| **2012** |  | 0.7 (0.5–0.8) | 0.9 (0.8–1.0) | 0.8 (0.7–0.8) | 0.7 (0.6–0.8) |
| **2013** | 1.0 (0.7–1.5) | 0.6 (0.5–0.8) | 0.9 (0.8–1.0) | 0.8 (0.8–0.8) | 0.7 (0.6–0.8) |
| **2014** | 0.7 (0.4–1.1) | 0.5 (0.4–0.7) | 0.9 (0.8–1.0) | 0.8 (0.7–0.8) | 0.6 (0.5–0.7) |
| **2015** | 1.0 (0.7–1.4) | 0.6 (0.5–0.8) | 0.9 (0.8–1.0) | 0.8 (0.8–0.9) | 0.6 (0.6–0.7) |
| **2016** | 0.6 (0.4–1.0) | 0.5 (0.4–0.6) | 0.9 (0.8–1.0) | 0.8 (0.8–0.8) | 0.7 (0.6–0.8) |
| **2017** | 1.1 (0.8–1.6) | 0.6 (0.5–0.8) | 0.9 (0.8–1.0) | 0.8 (0.8–0.9) | 0.7 (0.6–0.8) |
| **2018** | 0.9 (0.6–1.2) | 0.6 (0.5–0.7) | 1.0 (0.9–1.2) | 0.9 (0.8–0.9) | 0.7 (0.6–0.8) |
| **2019** | 0.8 (0.6–1.2) | 0.6 (0.5–0.7) | 1.1 (0.9–1.2) | 0.9 (0.8–0.9) | 0.7 (0.6–0.8) |
| **Pre-COVID-19 Average AAMR** | 1.26 (0.9–1.9) | 0.67 (0.5–0.9) | 1.07 (1.6–1.8) | 0.8 (0.8–0.8) | 0.66 (0.6–0.8) |
| **2020** | 2.2 (1.8–2.8) | 1 (0.9–1.2) | 1.7 (1.6–1.8) | 1.5 (1.4–1.5) | 2.6 (2.5–2.8) |
| **2021** | 5.9 (4.9–6.8) | 2.7 (1.9–3.7) | 2.9 (2.7–3.1) | 2.6 (2.6–2.7) | 4.1 (3.9–4.3) |
| **During COVID, Average AAMR** | 4.05 (3.35–4.8) | 1.85 (1.4–2.45) | 2.3 (2.15–2.45) | 2.05 (2–2.1) | 3.35 (3.2–3.55) |

**Table S4: Pneumothorax-related Average Annual Percentage Change Rate for NH Black or African American, NH White, NH Asian Pacific, NH American Indians, and Hispanic or Latino in the United States before and during the COVID-19 pandemic.**

| **Cohort** | **Range** | **AAPC** | **Lower CI** | **Upper CI** |
| --- | --- | --- | --- | --- |
| **NH American Indian or Alaska Native** | 1999-2019 | -3.2661 | -6.9404 | 0.5532 |
| **NH American Indian or Alaska Native** | 2019- 2021 | 169.7402* | 144.2188 | 197.9286 |
| **NH Asian or Pacific Islander** | 1999-2019 | -1.9541* | -2.8088 | -1.0919 |
| **NH Asian or Pacific Islander** | 2019- 2021 | 112.132 | -79.5037 | 2095.5224 |
| **NH Black or African American** | 1999-2019 | -0.0048 | -0.0627 | 0.0532 |
| **NH Black or African American** | 2019- 2021 | 64.8226* | 15.9904 | 134.2132 |
| **NH White** | 1999-2019 | 0.0232 | -0.5483 | 0.5979 |
| **NH White** | 2019- 2021 | 71.1173* | 48.6455 | 96.9862 |
| **Hispanic or Latino** | 1999-2019 | 2.6744* | 1.8268 | 3.5291 |
| **Hispanic or Latino** | 2019- 2021 | 151.673 | -47.5431 | 1107.4549 |

**Note: asterisk indicates p-value < 0.05**

**Table S5: Pneumothorax-related age-adjusted mortality rate for different age groups in the United States before and during the COVID-19 pandemic region**

|  | **Age-Adjusted Rates( 95% CI)** | | |
| --- | --- | --- | --- |
| **Years** | **Age 25 to 44 Years** | **Age 45 to 64 Years** | **Age≥ 65Years** |
| **1999** | 0.2 (0.2–0.2) | 0.9 (0.8–1) | 5.5 (5.3–5.8) |
| **2000** | 0.2 (0.1–0.2) | 0.9 (0.8–1) | 5.2 (5–5.5) |
| **2001** | 0.2 (0.1–0.2) | 0.8 (0.7–0.8) | 5 (4.8–5.2) |
| **2002** | 0.2 (0.1–0.2) | 0.7 (0.7–0.8) | 5.1 (4.9–5.3) |
| **2003** | 0.2 (0.1–0.2) | 0.7 (0.7–0.8) | 4.8 (4.6–5) |
| **2004** | 0.2 (0.1–0.2) | 0.6 (0.6–0.7) | 4.6 (4.4–4.8) |
| **2005** | 0.2 (0.1–0.2) | 0.8 (0.7–0.8) | 4.7 (4.5–5) |
| **2006** | 0.2 (0.1–0.2) | 0.7 (0.6–0.7) | 4.4 (4.1–4.6) |
| **2007** | 0.2 (0.1–0.2) | 0.7 (0.6–0.7) | 4.4 (4.2–4.6) |
| **2008** | 0.2 (0.1–0.2) | 0.7 (0.6–0.7) | 4.4 (4.2–4.6) |
| **2009** | 0.2 (0.1–0.2) | 0.7 (0.6–0.8) | 4.1 (3.9–4.3) |
| **2010** | 0.1 (0.1–0.1) | 0.7 (0.6–0.8) | 4.2 (4–4.4) |
| **2011** | 0.1 (0.1–0.1) | 0.7 (0.6–0.7) | 4.3 (4.1–4.5) |
| **2012** | 0.1 (0.1–0.1) | 0.7 (0.6–0.7) | 4.5 (4.3–4.7) |
| **2013** | 0.2 (0.1–0.2) | 0.7 (0.6–0.7) | 4.6 (4.4–4.8) |
| **2014** | 0.2 (0.1–0.2) | 0.7 (0.7–0.8) | 4.4 (4.2–4.6) |
| **2015** | 0.2 (0.1–0.2) | 0.7 (0.7–0.8) | 4.7 (4.5–4.9) |
| **2016** | 0.2 (0.1–0.2) | 0.8 (0.7–0.8) | 4.4 (4.3–4.6) |
| **2017** | 0.2 (0.1–0.2) | 0.8 (0.8–0.9) | 4.8 (4.6–5) |
| **2018** | 0.2 (0.1–0.2) | 0.8 (0.8–0.9) | 4.9 (4.7–5.1) |
| **2019** | 0.2 (0.1–0.2) | 0.9 (0.8–0.9) | 4.8 (4.7–5) |
| **Pre-COVID-19 Average AAMR** | 0.18 (0.10–0.18) | 0.74 (0.68–0.8) | 4.65 (4.46–4.87) |
| **2020** | 0.3 (0.3–0.3) | 1.7 (1.6–1.8) | 7.7 (7.5–8) |
| **2021** | 1 (1–1.1) | 4.3 (4.2–4.5) | 11.8 (11.6–12.1) |
| **During -COVID-19 Average AAMR** | 0.65 (0.65–0.7) | 3 (2.9–3.15) | 9.75 (9.55–10.05) |

**Table S6: Pneumothorax-related Average Annual Percentage Change Rate for different age groups in the United States before and during the COVID-19 pandemic**

| **Cohorts** | **Range** | **AAPC** | **Lower CI** | **Upper CI** |
| --- | --- | --- | --- | --- |
| **Age 25 to 44 Years** | 1999-2019 | -0.1534 | -1.1695 | 0.8731 |
| **Age 25 to 44 Years** | 2019 - 2021 | 156.1883* | 115.1139 | 469.0903 |
| **Age 45 to 64 Years** | 1999-2019 | -0.0977 | -0.8854 | 0.6964 |
| **Age 45 to 64 Years** | 2019 - 2021 | 88.8909* | 88.5922 | 89.1662 |
| **Age≥ 65Years** | 1999-2019 | -0.5366* | -1.0298 | -0.041 |
| **Age≥ 65Years** | 2019 - 2021 | 56.4143* | 53.2403 | 59.9157 |

**Note: asterisk indicates p-value < 0.05**

**Table S7: Pneumothorax-related Average Annual Percentage Change Rate for different census regions in the United States before and during the COVID-19 pandemic**

| **Age Adjusted Rate (95% CI)** | | | | |
| --- | --- | --- | --- | --- |
| **YEAR** | **NORTHEAST** | **MIDWEST** | **SOUTH** | **WEST** |
| **1999** | 1 (0.9–1.1) | 1 (0.9–1.1) | 1.2 (1.1–1.2) | 0.9 (0.8–1) |
| **2000** | 1 (0.9–1) | 0.9 (0.8–0.9) | 1.1(1-1.1) | 0.8 (0.7–0.9) |
| **2001** | 0.7 (0.7–0.8) | 0.9 (0.8–0.9) | 1 (0.9–1.1) | 0.8 (0.8–0.9) |
| **2002** | 0.8 (0.7–0.9) | 0.9 (0.8–1) | 1 (0.9–1.1) | 0.8 (0.8–0.9) |
| **2003** | 0.7 (0.7–0.8) | 0.8 (0.7–0.9) | 1 (0.9–1.1) | 0.7 (0.7–0.8) |
| **2004** | 0.8 (0.7–0.8) | 0.8 (0.7–0.8) | 0.9 (0.8–0.9) | 0.8 (0.7–0.8) |
| **2005** | 0.8 (0.8–0.9) | 0.8 (0.7–0.9) | 0.9 (0.9–1) | 0.8 (0.7–0.8) |
| **2006** | 0.7 (0.6–0.8) | 0.8 (0.7–0.8) | 0.9 (0.8–0.9) | 0.7 (0.7–0.8) |
| **2007** | 0.7 (0.6–0.8) | 0.7 (0.7–0.8) | 0.8 (0.8–0.9) | 0.7 (0.7–0.8) |
| **2008** | 0.7 (0.6–0.8) | 0.8 (0.7–0.8) | 0.9 (0.8–0.9) | 0.7 (0.6–0.7) |
| **2009** | 0.6 (0.5–0.7) | 0.7 (0.7–0.8) | 0.8 (0.8–0.9) | 0.8 (0.7–0.8) |
| **2010** | 0.6 (0.6–0.7) | 0.7 (0.6–0.8) | 0.9 (0.8–0.9) | 0.8 (0.7–0.8) |
| **2011** | 0.7 (0.6–0.7) | 0.7 (0.7–0.8) | 0.9 (0.8–0.9) | 0.7 (0.6–0.8) |
| **2012** | 0.7 (0.6–0.8) | 0.7 (0.7–0.8) | 0.8 (0.8–0.9) | 0.8 (0.7–0.8) |
| **2013** | 0.7 (0.6–0.7) | 0.7 (0.7–0.8) | 0.9 (0.8–0.9) | 0.8 (0.7–0.8) |
| **2014** | 0.7 (0.6–0.8) | 0.7 (0.7–0.8) | 0.9 (0.8–0.9) | 0.7 (0.7–0.8) |
| **2015** | 0.7 (0.6–0.8) | 0.9 (0.8–0.9) | 0.9 (0.8–0.9) | 0.8 (0.7–0.8) |
| **2016** | 0.7 (0.6–0.7) | 0.8 (0.7–0.8) | 0.9 (0.9–1) | 0.8 (0.7–0.8) |
| **2017** | 0.7 (0.6–0.7) | 0.8 (0.8–0.9) | 1 (0.9–1) | 0.8 (0.8–0.9) |
| **2018** | 0.7 (0.6–0.8) | 0.9 (0.8–0.9) | 1 (1–1.1) | 0.8 (0.8–0.9) |
| **2019** | 0.6 (0.6–0.7) | 0.9 (0.8–1) | 1 (1–1.1) | 0.8 (0.8–0.9) |
| **Pre-COVID Average AAMR** | 0.7 (0.6–0.8) | 0.8 (0.7–0.8) | 0.9(0.8–0.9) | 0.7(0.7–0.8) |
| **2020** | 1.1 (1.1–1.2) | 1.5 (1.4–1.5) | 1.7 (1.7–1.8) | 1.3 (1.3–1.4) |
| **2021** | 1.6 (1.5–1.7) | 2.3 (2.2–2.4) | 3.7 (3.6–3.8) | 2.8 (2.7–2.9) |
| **During-COVID Average AAMR** | 1.35 (1.3–1.45) | 1.9 (1.8–1.95) | 2.7 (2.65–2.8) | 2.05 (2–2.15) |

**Table S8: Pneumothorax-related Average Annual Percentage Change Rate for different Census regions in the United States before and during the COVID-19 pandemic**

| **Cohort** | **Range** | **AAPPC** | **Lower CI** | **Upper CI** |
| --- | --- | --- | --- | --- |
| **Northeast** | 1999-2019 | -1.7036* | -2.4793 | -0.9218 |
| **Northeast** | 2019-2021 | 56.4781 | -34.7145 | 275.0515 |
| **Midwest** | 1999-2019 | -0.4846 | -1.2606 | 0.2976 |
| **Midwest** | 2019-2021 | 66.6667* | 66.6614 | 66.6719 |
| **South** | 1999-2019 | -0.6661 | -1.355 | 0.0277 |
| **South** | 2019-2021 | 70.0000* | 69.9771 | 70.0229 |
| **West** | 1999-2019 | -0.1303 | -0.637 | 0.379 |
| **West** | 2019-2021 | 62.5000* | 62.4761 | 62.5239 |

**Table S9: Pneumothorax-related Average Annual Percentage Change Rate for different States in the United States before and during the COVID-19 pandemic**

| **STATES** | **1999-2019** | **2020-2021** |
| --- | --- | --- |
| **Alabama** | 1.1 (1–1.1) | 2.9 (2.6–3.2) |
| **Alaska** | 0.8 (0.6–1) | 1.3 (0.8–2.1) |
| **Arizona** | 0.7 (0.6–0.7) | 2.6 (2.3–3) |
| **Arkansas** | - 1. (1–1.2) | 2.6 (2.3–3) |
| **California** | 0.8 (0.8–0.8) | 2.1 (2–2.2) |
| **Colorado** | 0.7 (0.6–0.7) | 2.6 (2.3–2.9) |
| **Connecticut** | 0.7 (0.7–0.8) | 1.1 (0.9–1.3) |
| **Delaware** | - 1. (1–1.2) | 1.6 (1.1–2.2) |
| **District of Columbia** | 1 (0.8–1.1) | 1.9 (0.9–2.2) |
| **Florida** | 0.7 (0.7–0.7) | 2.2 (2.1–2.3) |
| **Georgia** | 0.9 (0.8–0.9) | 2.5 (2.3–2.7) |
| **Hawaii** | 1 (0.9–1.1) | 1.2 (0.8–1.6) |
| **Idaho** | 0.6 (0.6–0.7) | 2.1 (1.6–2.5) |
| **Illinois** | 0.7 (0.7–0.8) | 1.2 (1.1–1.3) |
| **Indiana** | 1 (0.9–1) | 2.8 (2.5–3.1) |
| **Iowa** | 0.7 (0.6–0.7) | 1.8 (1.5–2.1) |
| **Kansas** | 0.8 (0.7–0.8) | 2.4 (2–2.7) |
| **Kentucky** | - 1. (1.2–1.4) | 4.1 (3.7–4.5) |
| **Louisiana** | - 1. (1.2–1.4) | 1.5 (1.3–1.7) |
| **Maine** | 0.7 (0.7–0.8) | 0.5 (0.3–0.8) |
| **Maryland** | 1 (0.9–1) | 1.8 (1.6–2) |
| **Massachusetts** | 0.7 (0.7–0.8) | 1.1 (0.9–1.3) |
| **Michigan** | 0.8 (0.8–0.8) | 1.8 (1.6–2) |
| **Minnesota** | 0.7 (0.6–0.7) | 1.5 (1.3–1.7) |
| **Mississippi** | 0.9 (0.8–1) | 2.7 (2.3–3.1) |
| **Missouri** | 0.9 (0.9–1) | 2.4 (2.2–2.7) |
| **Montana** | 0.6 (0.5–0.7) | 2 (1.5–2.6) |
| **Nebraska** | 0.8 (0.7–0.9) | 2.6 (2.1–3.1) |
| **Nevada** | 1 (0.9–1.1) | 2.7 (2.3–3.1) |
| **New Hampshire** | 0.6 (0.5–0.7) | 1 (0.7–1.3) |
| **New Jersey** | 0.8 (0.7–0.8) | 2 (1.8–2.2) |
| **New Mexico** | 0.7 (0.6–0.8) | 2.3 (1.9–2.7) |
| **New York** | 0.6 (0.6–0.6) | 1.1 (1–1.2) |
| **North Carolina** | 0.9 (0.9–0.9) | 2 (1.8–2.2) |
| **North Dakota** | 0.8 (0.7–0.9) | 2.2 (1.5–3) |
| **Ohio** | 1 (0.9–1) | 2.2 (2–2.3) |
| **Oklahoma** | 0.9 (0.9–1) | 2.8 (2.5–3.2) |
| **Oregon** | 0.7 (0.6–0.7) | 1.5 (1.3–1.7) |
| **Pennsylvania** | 0.8 (0.8–0.8) | 1.7 (1.6–1.9) |
| **Rhode Island** | 0.9 (0.8–1) | 1.6 (1.1–2.1) |
| **South Carolina** | 1.1 (1–1.2) | 3 (2.7–3.4) |
| **South Dakota** | 0.7 (0.6–0.9) | 2.7 (2–3.5) |
| **Tennessee** | 1.2 (1.1–1.2) | 3.2 (2.9–3.5) |
| **Texas** | 1 (1–1.1) | 3.9 (3.8–4.1) |
| **Utah** | 0.5 (0.5–0.6) | 2.1 (1.7–2.4) |
| **Vermont** | 0.8 (0.6–0.9) | 1.1 (0.7–1.8) |
| **Virginia** | 0.8 (0.7–0.8) | 1.7 (1.5–1.9) |
| **Washington** | 0.9 (0.9–1) | 2 (1.8–2.2) |
| **West Virginia** | 1.4 (1.3–1.5) | 3.2 (2.7–3.7) |
| **Wisconsin** | 0.6 (0.5–0.6) | 1.4 (1.2–1.6) |
| **Wyoming** | 0.8 (0.7–1) | 3.3 (2.4–4.4) |
| **Total AAMRS** | 0.84(0.76-0.90) | 2.1 (1.7–2.4) |
|  |  |  |
